# Microenvironment-derived ADAM28 prevents cancer dissemination

**DOI:** 10.18632/oncotarget.26449

**Published:** 2018-12-14

**Authors:** Catherine Gérard, Céline Hubeau, Oriane Carnet, Marine Bellefroid, Nor Eddine Sounni, Silvia Blacher, Guillaume Bendavid, Markus Moser, Reinhard Fässler, Agnès Noel, Didier Cataldo, Natacha Rocks

**Affiliations:** ^1^ Laboratory of Tumor and Development Biology, GIGA-Cancer and GIGA-I3, GIGA-Research, University of Liege, Liege, Belgium; ^2^ ENT Department, University Hospital of Liege, Liege, Belgium; ^3^ Max-Planck-Institute of Biochemistry, Department of Molecular Medicine, Martinsried, Germany; ^4^ Department of Respiratory Diseases, CHU Liege and University of Liege, Liege, Belgium

**Keywords:** ADAM28, lung, metastasis, CD8^+^, T lymphocytes

## Abstract

Previous studies have linked cancer cell-associated ADAM28 expression with tumor progression and metastatic dissemination. However, the role of host-derived ADAM28 in cancer dissemination processes remains unclear.

Genetically engineered-mice fully deficient for ADAM28 unexpectedly display increased lung colonization by pulmonary, melanoma or breast tumor cells. In experimental tumor cell dissemination models, host ADAM28 deficiency is further associated with a decreased lung infiltration by CD8^+^ T lymphocytes. Notably, naive ADAM28-deficient mice already display a drastic reduction of CD8^+^ T cells in spleen which is further observed in lungs. Interestingly, *ex vivo* CD8^+^ T cell characterization revealed that ADAM28-deficiency does not impact proliferation, migration nor activation of CD8^+^ T cells. Our data highlight a functional role of ADAM28 in T cell mobilization and point to an unexpected protective role for host ADAM28 against metastasis.

## INTRODUCTION

ADAM28 (A Disintegrin And Metalloproteinase 28) is a membrane-bound proteinase (mADAM28) that also exists as a shorter secreted form (sADAM28) [1]. In humans, ADAM28 is mainly found to be expressed by lymphocytes, lymphoid tissues and epithelial cells whereas in mice, ADAM28 is expressed by epididymis and thymic epithelial cells. Both isoforms exhibit pro-tumor effects through their proteolytic activities [2, 3, 4, 5]. Overexpression of ADAM28 by carcinoma cells has been described in a wide range of cancers including breast cancer, chondrosarcoma [6], head and neck carcinoma [7], B-cell acute lymphoblastic leukemia [8], bladder carcinoma [9], non-small cell lung carcinoma (NSCLC) [10] and prostate cancer [11]. In NSCLC, ADAM28 expression levels correlate with cell proliferation, tumor growth and lymph node metastasis [10]. Yet, ADAM28 has been proposed as an effective biomarker for diagnosis and monitoring of NSCLC [12]. Several *in vivo* mouse studies demonstrated that specific knockdown of both ADAM28 forms in tumor cells using siRNA or shRNA delays primary tumor growth and lung metastasis of mammary MDA-MB-231 xenografts. Moreover, lung metastasis induced by an intravenous injection of prostatic PC-9 or MDA-MB-231 cells was decreased when ADAM28 expression was inhibited in tumor cells. Altogether, these findings strongly suggest that pro-tumor roles should be ascribed to ADAM28 produced by tumor cells and recommend ADAM28 as a promising therapeutic target.

Metastatic dissemination is not only dependent on intrinsic properties of tumor cells since the active participation of the surrounding tumor microenvironment, notably composed of endothelial cells, fibroblasts and immune cells, has been demonstrated. Hence, the adaptive immune system provides an effective response against tumors, mainly mediated by CD8^+^ cytotoxic T lymphocytes (CTLs), which induce tumor cell death through perforin-Granzyme release or *via* Fas/FasL pathway [13]. CD4^+^ T cells also play critical roles in anti-tumor responses as they indirectly stimulate CD8^+^ T cells by secreting pro-inflammatory cytokines that support CD8^+^ T cell activities [14]. In this context, infiltration of human lung tumors by CTLs has been significantly correlated with an improved outcome of NSCLC patients [15–21]. Moreover, predominant roles in restraining tumor growth and progression have been assigned to CTLs using transgenic mouse models of lung tumors.

In the present study, we demonstrate a strong link between host ADAM28 deficiency and tumor cell colonization in lungs. To assess ADAM28 implication in metastatic processes, we developed a mouse strain fully deficient for ADAM28. Surprisingly, ADAM28 deletion resulted in an increased metastatic colonization of lung tissues after intravenous injection of pulmonary LLC, melanoma B16K1 or mammary 4T1 cells. This induced tumor cell implantation in ADAM28 deficient lungs was associated with a strong reduction of CD8^+^ T cell infiltration in lung tissues as compared to wild-type (WT) animals. Our innovative data pinpoint a new protective role of host-derived ADAM28 against tumor cell colonization in lungs, specifically by its effects on cytotoxic CD8^+^ T cell mobilization to lungs bearing metastatic tumor islets, where CD8^+^ T cells are supposed to exert anti-tumor functions.

## RESULTS

### Generation and characterization of an ADAM28 full knockout mouse strain

**Figure 1:**
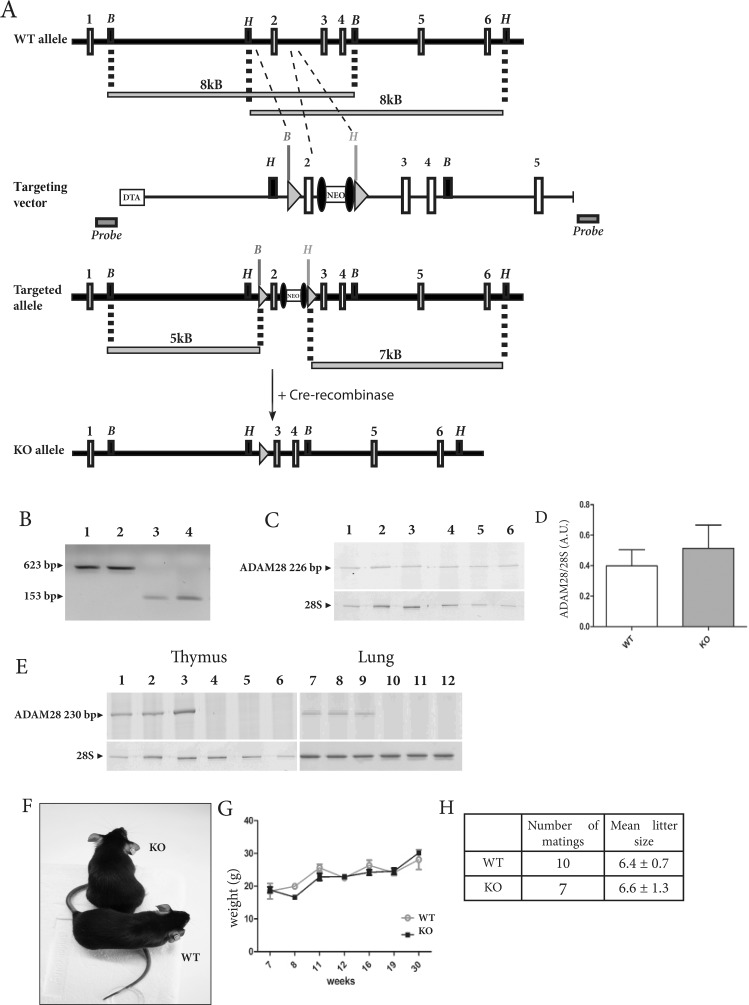
Knockdown strategy of the gene encoding ADAM28 in mouse **(A)** Targeting strategy to generate ADAM28 floxed (targeted allele) and ADAM28 null (KO allele) alleles. Restriction map depicting the wild-type (WT) ADAM28 gene locus (exons 1-6 (represented by white boxes)). In the targeting vector, the exon 2 has been flanked by loxP sites (grey triangles) and a neomycin selection cassette (NEO) flanked by frt sites (black ellipses). A diphteria toxin A (DTA) cassette is inserted at the 5′-site of the targeting vector. The grey bars under the restriction maps indicate sizes of BamHI (B) and HindIII (H) digestion–derived restriction fragments detected in wild-type and recombinant loci. DNA probes for Southern blot analysis (grey boxes) were designed outside the targeting vector. The ADAM28 null allele (KO) is obtained after a first Flp-excision of the NEO cassette followed by a Cre-mediated excision of exon 2. **(B)** DNA genotyping of WT (lanes 1-2) and ADAM28 KO littermates (lanes 3-4). ADAM28 KO mice bear a 153 bp-DNA short copy, while WT mice bear a 623-bp DNA copy of the ADAM28 gene. **(C)** Semi-quantitative RT-PCR measuring ADAM28 transcripts in lungs of tumor-free WT (lanes 1-3) and ADAM28 KO (lanes 4-6) mice. 28S ribosomal RNA levels are shown as loading control. **(D)** Quantification of relative ADAM28 mRNA expression in lungs of WT and ADAM28 KO mice. Results are expressed as arbitrary units corresponding to the ADAM28/28S ratio. Bars represent SEM (n=3). **(E)** Semi-quantitative RT-PCR measuring ADAM28 mRNA transcripts in thymus and lungs of WT (lanes 1-3; 7-9) and ADAM28 KO (lanes 4-6; 10-12) mice using primers targeting exon 2. **(F)** Wild-type and ADAM28 knockout littermates at 8 weeks of age. **(G)** Weight-gain curves of WT and ADAM28 KO littermates. Bars represent SEM. **(H)** Number of matings and mean litter size ± SEM for each genotype. These values were determined by considering the number of births obtained over one year for one couple per genotype.

As illustrated in Figure 1A, a conditional floxed allele of the ADAM28 encoding gene was generated by flanking exon 2 with loxP sites (targeted allele). Intercrossing of mice bearing a floxed ADAM28 allele, first with a deleter flippase, then with a deleter-Cre strain removed the neomycin selection cassette and the loxP-flanked exon 2, respectively. Intercrossing of heterozygous mice led to a mouse strain fully deficient for ADAM28 (KO) as well as to corresponding wild-type littermates. Amplification of DNA extracted from mouse tail biopsies using selective primers flanking the 5′ and 3′ extremities of the floxed exon 2 (just before and after both inserted loxP sites) showed that ADAM28-deficient mice bear a shorter DNA copy (153 base pairs, lanes 3-4) when compared to corresponding wild-type mice (623 base pairs, lanes 1-2) (Figure 1B), confirming the lack of exon 2. Cre-mediated deletion of exon 2 did not affect ADAM28 mRNA transcript levels in lungs, as measured by semi-quantitative RT-PCR using primers designed to amplify the active site domain of ADAM28 (Figure 1C-1D). However, using primers specifically targeting exon 2 confirmed the exon 2 removal since no transcripts could be amplified in thymus and lungs of ADAM28 KO mice (Figure 1E, lanes 4-6; 10-12). ADAM28 deficient pups display no apparent defect (Figure 1F), gain weight in the same manner as WT littermates over 30 weeks (Figure 1G) and are fertile. Moreover, ADAM28 KO and corresponding WT mice intercrosses yield similar litter size (Figure 1H).

In order to verify whether other members of the ADAM family, known to have pro-tumor functions and implicated in thymocyte development and maturation, were upregulated to compensate ADAM28 deficiency, Western Blot analyses measuring ADAM10 and ADAM17 levels were performed on thymic protein extracts from WT and ADAM28 deficient mice (Supplementary Figure 1). No significant difference in ADAM 10 and 17 protein levels was observed between experimental groups, ruling out compensatory mechanisms for ADAM28 deficiency.

### ADAM28 deficient mice display increased tumor cell colonization in lungs

**Figure 2:**
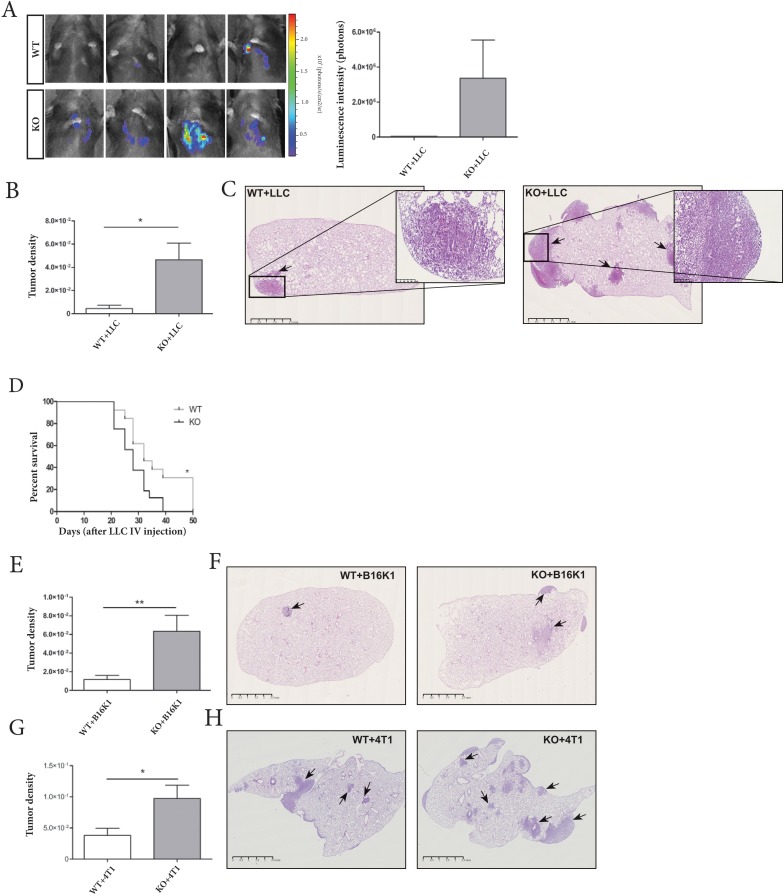
ADAM28 deletion in host tissues promotes metastasis to lung tissues **(A)** Biophotonic monitoring of lungs of WT (n=18) and ADAM28 KO mice (n=22), 21 days after intravenous injection of luciferase- transfected LLC cells. (Mann-Whitney; p=0.2). Images shown have been taken in ‘Photon mode’. The right panel shows the quantification of luminescent signals in regions of interest (ROI) in lungs. **(B)** Quantification of tumor density on HE-stained lung sections from WT (n=25) and ADAM28 KO (n=20) mice at day 21 after intravenous LLC cell injection. (Mann-Whitney; ^*^p< 0.05). Representative data from three independent experiments are shown. **(C)** Representative images of HE-stained lungs from WT and KO mice bearing LLC tumors (black arrows) (scale bar: left panel: 2500 μm). The right panel (scale bar: 250 μm) is a zoom of a tumor area present in lungs of each genotype. **(D)** Kaplan-Meier curves measuring survival of WT (n=13) and ADAM28 KO (n=17) mice over 50 days after intravenous LLC cell injection (Mantel-Cox; ^*^p=0.022). **(E)** Quantification of tumor density in HE-stained lung tissue slides of WT (n=20) and ADAM28 KO (n=19) mice at day 28 after B16K1 cell injection. (Mann-Whitney; ^**^p< 0.01). Representative data from three independent experiments are shown. **(F)** HE-stained lung sections from WT and KO mice bearing B16K1 tumors (arrow) (scale bar: 2500 μm) **(G)** Quantification of tumor density on HE-stained lung sections from WT (n=11) and ADAM28 KO (n=11) mice at day 14 after 4T1 cell injection. (Student's *t* test; ^*^p<0.05). Representative data from two independent experiments are shown. **(H)** Representative images of HE-stained lungs from WT and KO mice bearing 4T1 tumors (black arrows) (scale bar: 2500 μm).

To evaluate the implication of host-derived ADAM28 in metastatic processes, an experimental metastasis assay was conducted on ADAM28 deficient and corresponding control wild-type mice using intravenously injected luciferase-expressing LLC tumor cells. Interestingly, an increased LLC-related bioluminescence was quantified in lungs of ADAM28 deficient mice (p=0.2) (Figure 2A). Quantification of haematoxylin-eosin-stained histological lung sections confirmed a significant increase in tumor density in lungs of ADAM28 KO mice (KO+LLC) (^*^p<0.05) (Figure 2B-2C). Kaplan-Meier survival curves show that survival percentages of mice deficient for ADAM28 and injected with LLC cells were reduced, suggesting a more rapid metastatic pulmonary colonization (Figure 2D). The increased tumor cell dissemination to lung parenchyma of ADAM28 KO mice was also demonstrated with B16K1 melanoma cells (^**^p<0.01) (Figure 2E-2F). To verify whether the observed tumor phenotype was related to genetic background of the C57BL/6JRj mouse strain used in previous experiments, ADAM28 KO mice were backcrossed with Balb/cJRj mice and challenged with intravenous injections of mammary 4T1 cells. Again, Balb/cJRj ADAM28 KO mice displayed an enhanced tumor density in lungs when compared to corresponding WT littermates (^*^p<0.05) (Figure 2G-2H). Importantly, LLC, B16K1 and 4T1 tumor cells used in this study did not express ADAM28 mRNA (Supplementary Figure 2A), suggesting that only ADAM28 expressed by host non-tumoral tissues impacts tumor cell implantation to lungs. Taken together, these results demonstrate that ADAM28 deficiency in the pulmonary tumor-microenvironment is linked to increased tumor cell colonization in lungs.

### ADAM28 deficiency does not impact tumor cell proliferation and apoptosis

To evaluate the direct influence of ADAM28 deficiency on tumor cell behavior *in vivo*, proliferation and apoptosis rates were assessed in tumor foci colonizing lung tissues by Ki-67 and caspase 3 immunohistochemical staining, respectively. In this purpose, lungs of WT and ADAM28 KO mice bearing same sized-tumor islets were analyzed. As demonstrated in Supplementary Figure 2B-2C, the absence of ADAM28 in host tissues did not influence cell proliferation or apoptosis rates in tumors.

### ADAM28 deletion decreases CD8^+^ T cell recruitment to the spleen and to tumor-bearing lungs

**Figure 3:**
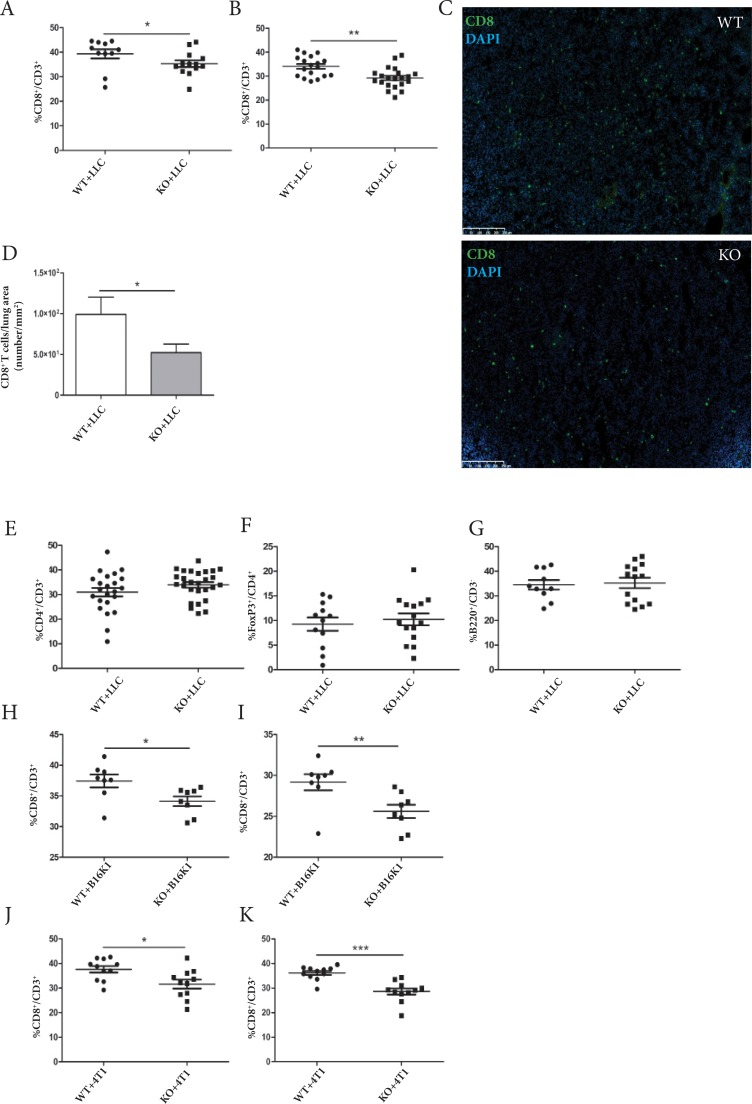
ADAM28 deficiency affects CD8^+^ T cell mobilization to splenic and pulmonary tissues in tumor-bearing mice **(A-B)** Percentages of CD8^+^/CD3^+^ T cell populations were evaluated in spleen (WT=11, KO=13) and lung tissues (WT=18, KO=21) from WT and ADAM28 KO mice 21 days following intravenous LLC cell injection (Student's *t* test; Mann-Whitney; spleen: ^*^p <0.05; lung: ^**^p <0.01). **(C)** Representative images of CD8^+^ T cells stained with anti-CD8 antibody (green) present in lungs bearing equally- sized tumors from WT and ADAM28 KO mice. Nuclei were stained with DAPI (blue). Scale bar: 250 μm. **(D)** Density of CD8 staining was measured and reported to total lung area of WT (n = 11) and ADAM28 KO (n = 15) mice (Mann-Whitney; ^*^p <0.05). **(E)** Evaluation of CD4^+^/CD3^+^ T cell population percentages by flow cytometry in lungs of WT (n=23) and ADAM28 KO (n=27) mice 21 days following intravenous LLC cell injection. **(F)** Evaluation of Treg (FoxP3^+^/CD4^+^) percentages in lungs of WT (n=12) and ADAM28 KO (n=15) mice at day 21 post-injection of LLC cells. **(G)** Percentages of B cells (B220^+^/CD3^−^) infiltrated within lungs of WT (n=10) and KO (n=14) mice 21 days after LLC cell injection. **(H-I)** Percentages of CD8^+^ T cells evaluated by flow cytometry on spleen and lungs from WT and KO (n = 8) at day 35 after B16K1 cell injection (spleen: Student's *t* test; ^*^p < 0.05) (lung: Mann-Whitney; ^**^p < 0.01). **(J-K)** Flow cytometry analysis performed on spleen and lungs from BALB/cJRj WT (n=11) and ADAM28 KO (n=11) mice 14 days following intravenous 4T1 cell injection. (Student's *t* test; spleen: ^*^p < 0.05; lung: ^***^p < 0.001).

As ADAM28 is described to be expressed by thymic epithelial cells and as CD8^+^ T cells are the main effectors in tumor immunity, the migration profiles of differentiated CD8^+^ T cells from the thymus to their secondary activation sites (spleen and lungs) were investigated. For this, flow cytometry analyses were performed on whole spleen and lungs derived from C57BL/6JRj WT and ADAM28 deficient mice, intravenously injected with LLC tumor cells (21 days after LLC cell injection), and from C57BL/6JRj tumor-free mice. Interestingly, among CD3^+^ cells present in splenic tissues, reduced CD8^+^ T cell percentages were observed in ADAM28 KO mice, 21 days after LLC cell injection (KO+LLC, ^*^p<0.05) (Figure 3A). A significant reduction of CD8^+^ T cell percentages was also noticed in ADAM28 KO lungs, once tumors were firmly established in the lung parenchyma (21 days after intravenous LLC cell injection) (^**^p<0.01) (Figure 3B). Immunofluorescence staining of CD8^+^ T cells infiltrating lungs bearing equally-sized tumors confirmed flow cytometry data showing a reduction of CD8^+^ T cell density in ADAM28 deficient lungs (Figure 3C-3D) (^*^p<0.05). Pulmonary infiltration by other T cell subtypes such as CD4^+^/CD3^+^ T cells, Treg (FoxP3^+^/CD4^+^) and B cells (B220^+^/CD3^−^) was not affected by ADAM28 depletion at day 21 post-injection (Figure 3E-3G).

As the increased metastatic burden observed in ADAM28 KO mice was corroborated using different cell lines (LLC, B16K1 and 4T1) and different mouse strains (C57BL/6JRj and Balb/cJRj), we investigated whether infiltration of CD8^+^/CD3^+^ T cells was also affected in C57BL/6JRj mice injected with B16K1 cells or 4T1-injected Balb/cJRj mice. Hence, spleen and lungs of C57BL/6JRj ADAM28 KO mice, injected with B16K1 cells and bearing well developed tumors, displayed significant lower CD8^+^ T cell percentages as compared to corresponding WT littermates (day 35 post-injection, ^*^p<0.05; ^**^p<0.01) (Figure 3H-3I). Decreased CD8^+^ T cell numbers were also found in spleen of ADAM28 knockout mice after intravenous 4T1 cell injection (Figure 3J) (^*^p<0.05). Moreover, a clear reduction of CD8^+^ T cell counts could be noticed in ADAM28 deficient lungs once 4T1 tumors were well established in lung tissues (14 days after 4T1 tumor cell injection) (^***^p<0.001) (Figure 3K).

**Figure 4:**
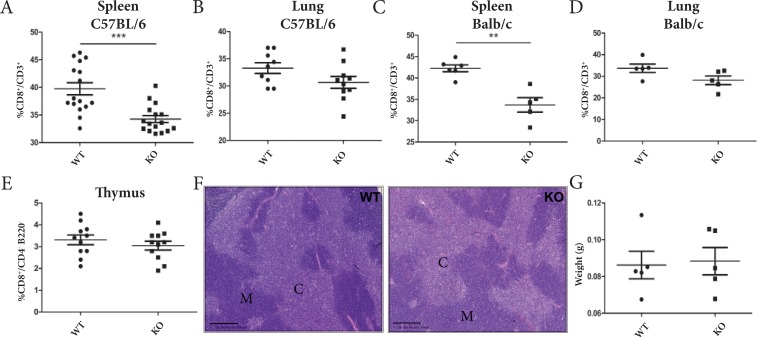
ADAM28 deficiency reduces CD8^+^ T cell mobilization to splenic and pulmonary tissues in tumor-free mice **(A-B)** CD8^+^/CD3^+^ T cell percentages assessed in spleen (n=16) and lungs (WT=9, KO=10) of tumor-free C57BL/6JRj WT and ADAM28 KO mice (Student's *t* test; ^***^p < 0.001). **(C-D)** Flow cytometry analyses were performed to evaluate CD8^+^/CD3^+^ cell populations present in spleen (WT=6, KO=5) and lungs (WT=5, KO=5) of tumor-free BALB/cJRj WT and ADAM28 KO mice (Mann-Whitney; ^**^p <0.01). **(E)** Percentages of CD8^+^ T cells (CD8^+^/CD4^−^ B220^−^) evaluated by flow cytometry in thymus of tumor-free WT (n=11) and ADAM28 KO (n=11) mice (Student's *t* test; p>0.05). **(F)** Representative HE-stained sections of thymus derived from WT and ADAM28 KO mice. Cortex (C) and medulla (M). Scale bar: 500 μm. **(G)** Thymus weight of 6 week-old WT (n=5) and ADAM28 KO (n= 5) mice (Student's *t* test).

Taken together, our data show that the observed loss in CD8^+^ T cell numbers is correlated with ADAM28 deficiency but is not dependent on the mouse strain (C57BL/6JRj or Balb/cJRj) or the tumor cell line used (LLC, B16K1 or 4T1) suggesting a conserved mechanism among different experimental models.

### ADAM28 deficiency does not alter thymus development

No difference of thymocyte differentiation stages (double-negative CD4^−^/CD8^−^ (DN1-4); double-positive CD4^+^/CD8^+^ (DP) (Supplementary Figure 4) or mature single positive cells (CD4^+^/CD8^−^/B220^−^ and CD8^+^/CD4^−^/B220^−^) (Figure 4E, Supplementary Figure 4) could be highlighted in thymus of 8 week-old tumor-free WT and ADAM28 KO mice. The histological organization of thymus is similar between both experimental groups as demonstrated on thymic tissue slides where cortex (C) and medulla (M) distribution is comparable (Figure 4F). Thymus weight was also not affected by ADAM28 deficiency (Figure 4G).

Altogether, these results demonstrate that ADAM28 KO mice display reduced CD8^+^ T cell percentages in spleen that are not linked to an impaired upstream T cell maturation in thymic tissues. Hence, the downstream reduced infiltration of CD8^+^ T cells in tumor-bearing ADAM28 KO lungs and the putative consequent reduction of CD8-related anti-tumor activity seem to promote colonization of tumor cells in pulmonary tissues.

### ADAM28 deficiency does not impair CD8^+^ T cell activity, proliferation and migration

**Figure 5:**
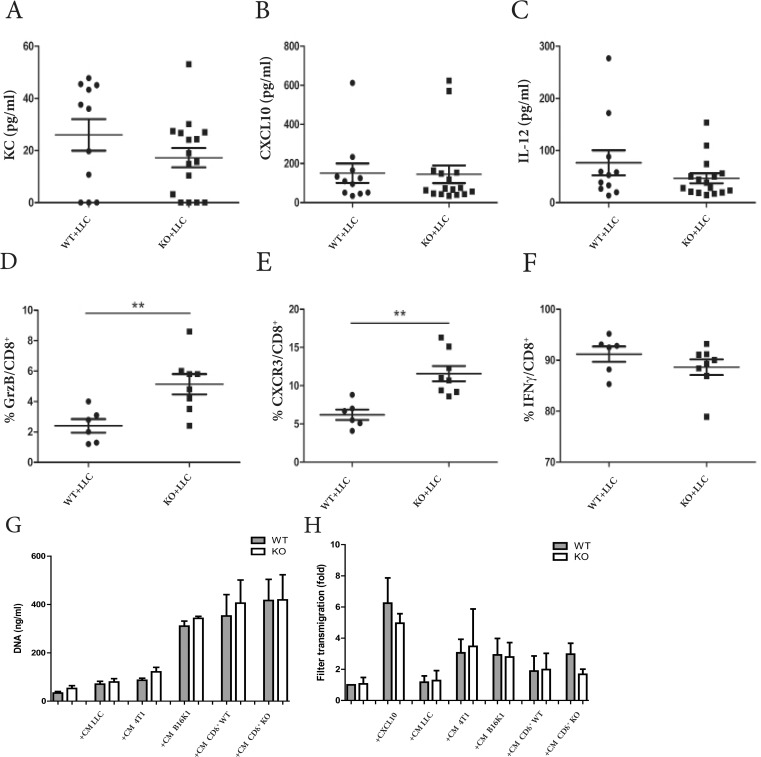
Functional features of ADAM28 deficient CD8^+^ T cells are not impaired **(A-C)** Production of KC **(A)**, CXCL10 **(B)** and IL-12 **(C)** was assessed by ELISA on lung protein extracts from WT (n=11) and ADAM28 KO (n=16) mice bearing equally-sized tumors (Student's *t* test; Mann-Whitney; p>0.05). **(D-F)**. Quantification of Granzyme B (GrzB) **(D)**, CXCR3 **(E)** and IFN-γ **(F)** expression on CD8^+^ T cells by flow cytometry in total lung extracts of WT (n=6) and ADAM28 KO (n=8) mice after intravenous LLC cell injection (Day 21) (Student's *t* test; ^**^p < 0.01). **(G)** Proliferation assay performed on ADAM28 KO (n=6) and WT (n=6) CD8^+^ T cells stimulated by CM from tumor cells (LLC, 4T1, B16K1) or by CM from CD8^+^ T cells (Mann-Whitney). **(H)** Migration assay on CD8^+^ T cells isolated from WT (n=4) and ADAM28 KO (n=4) mice. Chemoattractants used in the lower chamber of the Boyden Chamber were either CXCL10, CM from tumor cells (LLC, 4T1, B16K1) or CM from CD8^+^ T cells previously isolated from WT or ADAM28 deficient mice (Mann-Whitney). All results were normalized to the migration of WT CD8^+^ T cells (without stimulation) considered as baseline migration and expressed as fold increase from baseline.

Proliferation of CD8^+^ T cells is correlated with their activation status. Hence, an altered ability to respond to activating stimuli could explain lower numbers of infiltrating CD8^+^ T cells observed in spleen and tumor-bearing lungs of ADAM28 deficient mice. To investigate whether the activity profile of CD8^+^ T cells was impaired by ADAM28 deficiency, expression levels of cytokines involved in regulating CD8^+^ T cell responses were quantified on whole lung protein extracts from ADAM28 deficient and corresponding WT mice bearing equally-sized metastatic foci in lungs. No difference was observed for KC and CXCL10 between experimental groups (Figure 5A-5B). Levels of IL-12, which directly induce CD8^+^ T cell activation, expansion and proliferation were not altered by ADAM28 deletion indicating that the ADAM28 deficient microenvironment has no effect on the release of soluble factors known to regulate CD8^+^ T cell responses (Figure 5C).

Interestingly, antigens specific to CD8^+^ T cell activation such as Granzyme B (GrzB) or CXCR3 were significantly higher in lungs of ADAM28 KO mice (^**^p<0.01) (Figure 5D-5E), 21 days after intravenous LLC cell injection. These increased Granzyme B and CXCR3 levels observed in ADAM28 deficient lungs can thus be associated with the increased lung tumor density. Moreover, CXCR3 levels expressed on CD8^+^ T cells isolated from tumor-free WT and ADAM28 KO mice and stimulated *ex-vivo* with IL-2 and IL-7 were similar between both groups (Supplementary Figure 5A). Levels of IFN-γ, which plays a critical role in control of CD8^+^ T cell expansion, were similar between experimental groups (Figure 5F). These results illustrate that CD8^+^ T cells infiltrating tumor-bearing lungs are well activated even if ADAM28 is lacking.

In addition, basal proliferation rates were similar among CD8^+^ T cells isolated from naive WT and ADAM28 KO spleens. Stimulation of CD8^+^ T cells with media conditioned (CM) either from LLC, 4T1 or B16K1 tumor cells or from wild-type or from ADAM28 deficient CD8^+^ T cells induced their proliferation, regardless of ADAM28 production (Figure 5G). Migration of CD8^+^ T cells purified from spleens of ADAM28 KO or WT mice was assessed by modified Boyden Chamber assays using CXCL10 at different concentrations (Figure 5H, Supplementary Figure 5B), CM from tumor cells or CM from CD8^+^ T cells used as chemoattractants (Figure 5H). Similar profiles of migration have been observed between ADAM28 KO and WT CD8^+^ T cells, regardless the chemoattractants used (Figure 5H). Cytotoxic effects of *ex vivo*-isolated CD8^+^ T cells were also assessed and no difference of death or apoptosis rates of LLC cells was observed (Supplementary Figure 5C). These data demonstrate that CD8^+^ T cells isolated from ADAM28 KO mice display similar proliferation, migration and cytotoxic abilities as their WT counterparts. Altogether, these results demonstrate that cytokines involved in activation and stimulation of CD8^+^ T cells are commonly produced in an ADAM28-depleted tumor-microenvironment.

## DISCUSSION

Previous reports, focusing on cancer cell-derived ADAM28, have demonstrated that ADAM28 promotes cancer development and progression [22, 23]. In the present study, the influence of total ADAM28 depletion in mouse tissues was evaluated on physiological and metastatic processes. For the first time, we demonstrate a protective role of stromal cell-derived ADAM28 against colonization of tumor cells to lung tissues. This exaggerated cancer cell dissemination in ADAM28 deficient animals is associated with a defect in CD8^+^ T cell trafficking to tumor-bearing organs. These findings are supported by robust experimental data: 1) an increased metastatic burden observed in lungs of ADAM28 KO mice, without regard to cancer cell type (pulmonary LLC, mammary 4T1, melanoma B16K1) or mouse strain (C57BL/6JRj and Balb/cJRj) used in experimental settings, 2) decreased CD8^+^ T lymphocyte counts in spleen as well as in lung tissues bearing well-developed tumors of ADAM28 KO animals, 3) the impaired CD8^+^ T cell recruitment already evident in the spleen of tumor-free ADAM28 deficient animals.

Interestingly, tumor cells themselves are not affected by the depletion of host-derived ADAM28 since their proliferation and apoptosis rates were found to be similar in lungs of ADAM28 KO and WT animals.

T lymphocytes are the main effectors of anti-tumor immune responses and their abundance in tumors represents a precious prognostic factor for various cancers [24, 25, 26]. Hence depletion or inhibition of CD8^+^ T cells in experimental mouse models negatively impacts tumor progression outcomes [27, 28].

Development and maturation of T-cells is a complex multistep process, where bone marrow-derived T cell progenitors, reaching the thymus, undergo various maturation stages. ADAM28 has been shown to be expressed in both fetal and adult thymic epithelial cells, and its deficiency could thus lead to impaired thymus development. In this work, complete depletion of ADAM28 did not modify morphological development or functionality of the thymus and no defect of lymphocyte maturation processes was found. ADAM10 and ADAM17 levels were measured to corroborate whether a compensatory mechanism was implemented in animals lacking ADAM28, which could explain that lymphocyte maturation is not affected in our model. Indeed, ADAM10 and ADAM17 seem to be key molecules for the thymus development since their *in vivo* deficiency has been correlated to impaired thymocyte expansion and maturation. Our results demonstrate that ADAM10 and ADAM17 expressions were comparable between ADAM28 KO and WT samples, and further suggest that ADAM28 is not essential for T cell maturation in thymus.

ADAM28 could contribute to leukocyte transmigration by binding α_4_β_1_ and α_4_β_7_ integrins [29–31] and is overexpressed in lymphoblastic leukemia [32]. In addition, a recent study showed that ADAM28 promotes MZ B-cell generation by Notch2 cleavage [33]. In the present study, the distribution of lymphocytic population subtypes was evaluated within different organs implicated in T cell trafficking. While significant lower counts of CD8^+^ T cells were detected in spleen of ADAM28 KO mice, no difference of B, CD4^+^ T and Treg cell populations was found. Accordingly, we hypothesize that the observed decreased CD8^+^ T cell population in spleen impacts the proportion of CD8^+^ T cells infiltrating tumor-bearing lungs. To verify whether tumor density might have an influence on CD8^+^ T lymphocyte infiltration, CD8^+^ T cell pulmonary infiltration was compared between ADAM28 deficient and WT mice by specific CD8-immunofluorescence staining on equally-sized tumors and was also found to be decreased in lungs of ADAM28 KO mice.

Production of KC, CXCL10 and IL-12 cytokines, involved in CD8^+^ T cell infiltration and CD8^+^ T cell activity regulation [34], was explored within tumors but no difference of cytokine production was found between experimental groups. By various *ex vivo* assays, we demonstrated that intrinsic CD8^+^ T cell proliferation, which is closely linked to their activation status, as well as their migration abilities were not impaired in ADAM28 deficient animals. Moreover, an *ex vivo* short term cytotoxic assay could demonstrate that CD8^+^ T cells isolated from ADAM28 KO mice are able to efficiently kill tumor cells. Higher percentages of Granzyme B and CXCR3 were identified on ADAM28 KO CD8^+^ T cells isolated from tumor-bearing lungs, which can be correlated to the increased tumor density in ADAM28 KO lungs used for this assay. These results demonstrate that CD8^+^ T cells originating from ADAM28-deficient mice can be effectively activated *in vivo*.

ADAM and other matrix proteinases have been largely recommended in the literature as promising therapeutic targets to counteract cancer progression and metastasis spread. However, one has to be careful since these promising targets might display dual roles according to their expression pattern. Notably, it was previously reported that MMP12 increases tumor invasiveness only when expressed by tumor cells whereas its expression by macrophages can be associated with a better prognosis [35]. Moreover, in an experimental breast cancer mouse model, an enhanced tumor progression is correlated with ADAM12 overexpression by tumor cells that is not described when ADAM12 is expressed by stromal cells. On the other hand, ADAM12 deficiency in immortalized tumor cells isolated from PyMT mice suppresses breast tumor progression although no difference in tumor growth is observed between ADAM12 null mice and wild type littermates [36]. ADAMTS1 (ADAM with ThromboSpondin motifs 1), first suggested as an interesting therapeutic target in breast cancer management [37], has been defined several years later as playing a suppressive role in tumor development and was thus rather proposed as facilitating apoptotic and anti-tumor effects of adjuvant factors used in cancer treatment [38]. These contradictory roles seem to be depending on molecular properties of ADAM(TS) proteinases. Indeed, a study showed that ADAMTS1 impacts angiogenic signaling events through two distinct mechanisms. First, carboxyl-terminal domain of ADAMTS1 binds to VEGF and reduces VEGFR2 phosphorylation affecting the angiogenic pathway [39, 40]. Second, catalytic activity of ADAMTS1 is required to exert its proangiogenic effects as a single mutation in the metalloproteinase domain is associated with antiangiogenic/antitumor activities *in vivo* [40, 41]. ADAM23, a non-proteolytic ADAM, is reported to exert anti-tumoral effects through its disintegrin domain as it specifically interacts with α_V_β_3_ integrin. In MDA-MB435 cells, it was demonstrated that downregulation of ADAM23 enhanced α_V_β_3_ integrin activation leading to an increased adhesion and migration of these cells to classic integrin ligands. In addition, ADAM23 knockdown was associated with an enhanced pulmonary tumor cell arrest in immunodeficient mice [42].

Altogether, these studies highlight the relevance of both the metalloproteinase and the disintegrin domains of ADAM and ADAMTS proteinases. Since ADAM28 exhibits proteolytic activities, it could be interesting to further investigate these functions through the identification of new specific substrates. Indeed, the known substrates of ADAM28 are common with other ADAMs due to the highly conserved structure. For example, IGFBP-3 (Insulin-like growth factor binding protein-3) is also cleaved by ADAM12 and vWF (von Willebrand Factor) is also processed by ADAMTS13. In addition, the disintegrin domain could also be involved in important mechanisms as ADAM28 binds different integrins, namely α_4_β_1_, α_4_β_7_ and α_9_β_1_.

These examples underline the ambivalence concerning the role of metalloproteinases in processes leading to tumor development and increase the complexity of considering ADAMs as therapeutic targets.

Altogether, our results highlight the crucial importance of ADAM28 expressed by the stromal compartment in the control of cancer dissemination and demonstrate that ADAM28 deficiency decreases CD8^+^ T cell numbers. These findings strongly advocate considering ADAM28 as an anti-target protease and raise the question whether ADAM28 expression should be used as a prognostic factor for cancer patients.

## MATERIALS AND METHODS

### Cell culture

Luciferase-expressing mouse Lewis Lung Carcinoma (LLC) and mouse 4T1 breast cancer cells were purchased from Caliper Life Sciences (Hertogenbosch, Netherlands) and American Type Culture Collection (Molsheim, France), respectively. Murine melanoma B16K1 cells were used as previously described [43]. Cells were cultured in Dulbecco's modified Eagle's medium (DMEM, Gibco, Thermo Fisher Scientific, Merelbeke, Belgium) supplemented with 10% fetal bovine serum (FBS), penicillin-streptomycin (100 U/ml–100 μg/ml) and L-glutamine (2 mmol/l) and maintained at 37°C under 5% CO_2_ atmosphere and 95% humidity. Cells were authenticated by Leibniz-Institute DSMZ GmbH. Experiments were performed until maximum 10 cell passages after authentication. Conditioned media were obtained as described previously [44]. Briefly, cells were starved with serum-free DMEM and after 1 hour the medium was replaced with fresh serum-free DMEM. The conditioned medium was collected after 48 hours, centrifuged at 1200 rpm for 10 minutes and aliquots were stored at −80°C until use.

### Animals

Animals were housed in the animal facility of the University of Liege (Belgium) with respect to the Animal Ethical Committee rules. All animal experiments were approved under the references #1376, #1597, #1945 by the Animal Ethical Committee of the University of Liege in accordance with the institution guidelines for animal care.

### Generation of conditional ADAM28 knockout mice using the Cre-Lox recombination technology

To design a conditional knockout construct for ADAM28, mouse BAC DNA was purchased from Source Bioscience Lifescience and transfected into Rec+ bacteria (SW102). The targeting vector was designed by inserting loxP sites into introns around the exon 2, which will be removed after recombination by a deleter-Cre recombinase, leading to a reading frame shift and, consequently, to the appearance of an early stop codon in the exon 3 and an incomplete shorter protein.

Briefly, 5′ and 3′ annealing arms of the ADAM28 gene were introduced respectively before and after the loxP site and the frt-flanked neomycin selection cassette already present in the pL451-vector (Molecular Medicine Department, Max Planck Institute of Biochemistry, Munich, Germany) using EcoRV, ClaI, BamHI, and NotI restriction enzymes. The distal LoxP site and the exon 2 were then inserted by PCR in the pL451-vector using EcoRV and EcoRI enzymes. Correct insertion of all fragments was verified by DNA sequencing and enzymatic digestion. To ensure maximal accurate homologous recombination and avoid random genomic integration of the targeting construct, a diphtheria toxin A (DTA) cassette was inserted at the 5′-site of the targeting construct. The 5′ and 3′ arms corresponding to ADAM28 sequences in the pL451 plasmid were then used for recombination with corresponding sequences on BAC DNA. The final targeting construct was retrieved into pBluescript SK vector (Stratagene, San Diego, California) using the flanking homology arms on 5′ and 3′. The linearized targeting vector was then electroporated into mouse R1 embryonic stem cells, which were previously cultured on a feeder layer of ɣ-irradiated embryonic fibroblast cells [45]. After 24 hours without selection, transfected cells were selected with geneticin (G418, InvivoGen, San Diego, California). After 10 days, surviving clones were subcultured, then half of each colony was frozen while DNA was extracted from the other half to check for correct homologous recombination by Southern Blot analysis using external probes. For this, each colony was screened by performing BamHI or HindIII enzymatic digestions, yielding respectively 5kb and 7kb bands in targeted alleles (Figure 1A). At least 5 mutant stem cell clones were injected into C57BL/6 blastocysts, which were further transplanted into foster mice to generate germline chimeras. Chimeric mice were mated with C57BL/6 females to test for germline transmission. Chimeras derived from 4 different clones gave germline transmission. Chimeric mice were then first crossed with transgenic C57BL/6 mice carrying a deleter-flp recombinase (Thierry Van Reeth unpublished, #012930 The Jackson Laboratory, Maine, USA) to remove the neomycin selection cassette and further intercrossed with C57BL/6 mice containing a deleter-Cre recombinase (KO allele) [46] (#006054, The Jackson Laboratory). To obtain homozygous mice fully deficient for the ADAM28 gene, heterozygous mice were intercrossed and tail biopsies were assayed for DNA expression by PCR analysis to identify wild-type, heterozygous or ADAM28 knockout mice. Chimeric mice were backcrossed at least 10 times with C57BL/6JRj or Balb/cJRj mice prior to experimental procedures. Wild-type control mice used in each experiment are always littermates of corresponding ADAM28 knockout animals.

### Genotyping

Mouse genotyping was performed using PCR amplification of genomic DNA extracted from tail biopsies (NucleoSpin Tissue Kit, Macherey-Nagel, Hoerdt, France). PCR reactions were performed with a Taq Polymerase Kit (Takara Taq, Clontech, Saint-Germain-en-Laye, France) using primers that were designed just before and after the targeting construct: (F) 5′-GGAGAACTGGATTCTGCCAA-3′, and (R) 5′-TAGTTTCAGACGTGAGTGATCG-3′. PCR products were resolved on agarose gels.

### Experimental *in vivo* mouse models of metastatic dissemination

LLC, B16K1 and 4T1 cells (1×10^5^) were suspended in 100 μl of serum-free DMEM and intravenously (IV) injected in the lateral tail vein of ADAM28 KO and WT mice (C57BL/6JRj or Balb/cJRj). Lung metastasis was monitored by measuring bioluminescence of LLC cells up to 21 days after tumor cell injection, using *in vivo* Imaging System Xenogen IVIS 200® (Caliper Life Sciences) as described previously [47]. Mice injected with B16K1 and 4T1 cells, not transfected with the luciferase gene, were sacrificed 28 days and 14 days respectively after tumor cell injection. In another experimental setting, to accurately compare LLC lung tumors induced in ADAM28 KO and WT mice, metastatic dissemination was monitored in all mice every second day until a bioluminescent signal of 1.5×10^6^ relative to LLC tumor development was reached in Regions of Interest (ROI) determined around lung tissues. Tumor growth and physical well-being were monitored up to 50 days after tumor cell injection. Animals were euthanized by cervical dislocation at 1.5×10^6^ bioluminescent signal. At day 50, surviving mice were sacrificed. Kaplan-Meier tumor survival curves were established and P values were calculated with log-rank (Mantel-Cox) test.

### Flow cytometry

To evaluate T lymphocyte populations in WT and KO tissues, spleen, thymus and lungs were harvested and fibrotic tissues were digested in collagenase C (1mg/ml; Gibco) prior to red blood cell lysis (Red Blood Cell Lysis Buffer, Sigma Aldrich, Saint-Louis, Missouri). Recovered cells were washed and filtered through a 41 μm nylon net filter (Merck). Cells were then washed, stained with fluorochrome-conjugated surface antibodies during 30 minutes, fixed and permeabilized using Cytofix/Cytoperm (BD Biosciences, Erembodegem, Belgium) before intracellular antigen staining. For cytokine staining, cells (10^6^ cells/100μl) were stimulated for 4 hours (Leukocyte Activation Cocktail, with BD GolgiPlug, BD Pharmingen, Erembodegem, Belgium) prior staining with fluorochrome-conjugated antibodies. Following antibodies were used for flow cytometry analysis: CD45-V450 (30-F11), CD3-APC CY7 (17A2), CD8-PE (53-6.7), CD4-PERCP CY 5.5 (RM4-5), CD8a-BB515 (53-6.7), CD45R-APC-eFluor506 (B220) (RA3-6B2), CD183-APC (CXCR3-173), IFN-γ-PE CY7 (XMG 1.2), TCR-β-APC (H57-597), CD44-APC CY7 (IM7), CD45R-BV510 (B220) (RA3-6B2), CD25-BV421 (7D4) (BD Biosciences), FoxP3-PE (FJK-16s), Granzyme B-PE (NGZB), CD3-eFluor506 (17A2), CD45R-APC-eFluor780 (B220) (RA3-6B2) (eBioscience, Thermo Fisher Scientific). Data were acquired on FACS CANTO II flow cytometer (BD Biosciences) and analyzed using BD FACSDiva software (BD Biosciences).

### Tissue preparation

Histologic sections of paraffin-embedded lung and thymus tissues (5 μm) were stained with hematoxylin and eosin (HE) and imaged using the digital slide scanner NanoZoomer 2.0-HT system (0.46 μm/pixel (20X) scanning resolution) (Hamamatsu Photonics, Hamamatsu City, Japan). Metastatic dissemination to lungs was evaluated on five HE-stained sections (separated each other by 50 μm) per mouse lung by measuring tumor area in lungs and reporting it to the total area of lung tissue analyzed. Quantification of lung tumor area was performed as described previously [48].

### Immunofluorescence staining

Frozen lung sections (6 μm) were fixed in acetone at −20°C and in methanol 80% at 4°C before incubation with a primary rat anti-mouse CD8a antibody (BD Pharmingen). After washing, slides were incubated with goat anti-rat Alexa Fluor 488-conjugated secondary antibody (Invitrogen, Carlsbad, California) and mounted with DAPI Fluoromount G (SouthernBiotech, Birmingham, USA). Images were taken with the digital slide scanner NanoZoomer 2.0-HT system and LX2000 source (0.46 μm/pixel (20X) scanning resolution).

### Interleukin quantification by ELISA

For specific dosages of IL-12, IL-8 (KC), IL-10, CXCL10, DuoSet ELISA Development kits (R&D Systems, Minneapolis, Minnesota) were used following the manufacturer's instructions.

### CD8^+^ T cell isolation and expansion

CD8^+^ T cells were isolated from spleens of 8 week-old ADAM28 KO or WT mice as described previously [49]. Briefly, after lysis of red blood cells, cells were filtered through a 40 μm-cell strainer. CD8^+^ T cells were isolated using CD8a (Ly-2) MACS microbeads (Miltenyi Biotec, Bergisch Gladbach, Germany) according to the manufacturer's instructions. Isolated CD8^+^ T cells were then plated into 6-well plates previously coated with 0.5 μg/ml anti-CD3 and 5 μg/ml anti-CD28 antibodies. After 24 hours, recombinant mouse IL-7 (0.5 ng/ml) and IL-2 (0.2 ng/ml) (R&D Systems) were added to each well. After 4 days of culture, cells were harvested for further analyses. Conditioned medium was obtained as described above. For this, CD8^+^ T cells were cultured with serum-free RPMI for 48 hours. Conditioned medium was centrifuged and aliquots were stored at −80°C until use.

### Proliferation assay

Proliferation assay was performed using a CyQUANT cell proliferation assay kit (Invitrogen). Briefly, 4×10^3^ CD8^+^ T cells were seeded in each well of a 96-well plate and cultured either in RPMI medium supplemented with 2% FBS, in RPMI medium supplemented with medium conditioned by LLC, B16K1 or 4T1 tumor cells or in medium conditioned by CD8^+^ T cells previously isolated from ADAM28 KO or WT spleens. Culturing cells in serum-free RPMI medium assessed basal proliferation of CD8^+^ T cells. A cell-lysis buffer containing a green dye was added in each well and fluorescence intensity, reflecting cell proliferation, was measured using a SpectraMax I3 microplate reader (Molecular Devices, Sunnyvale, California).

### *Ex vivo* migration assay

Activated CD8^+^ T cells (2×10^5^ cells/200 μl) were suspended in RPMI supplemented with 0.1% BSA and placed in the upper chamber of a 6.5mm transwell insert displaying a 5 μm-pore size membrane (Corning, Corning, New-York). Conditioned medium recovered from tumor cells (LLC, B16K1, 4T1 cells) or from CD8^+^ T cells isolated from KO and WT spleens was added in the lower chamber. CXCL10 (100 nM, R×D Systems) present in the lower chamber of the Boyden Chamber was used as a positive control and migration of CD8^+^ T cells without chemoattractant was determined using serum-free RPMI medium. After 3 hours of incubation, CD8^+^ T cells, which migrated through the filter, were harvested and stained with anti-CD8a-PE antibody. CD8^+^ T cells were enumerated in each experimental condition during 1 minute by FACS CANTO II flow cytometer (BD Biosciences). Data were analyzed using BD FACSDiva software (BD Biosciences).

### RNA Extraction and Reverse Transcription–Polymerase Chain Reaction (RT-PCR)

Total RNAs were extracted from lungs using RNeasy Mini kit (Qiagen, Hilden, Germany) according to the manufacturer's protocol. RT-PCR was performed on 10 ng of total RNA using GeneAmp Thermostable rTth Reverse Transcriptase RNA PCR Kit (Roche Life Science, Indianapolis, Indiana). The following primers targeting respectively ADAM28 exon 11 and exon 2 were used: (F) 5′-CTACTTGAGCTGCAAGTGTCC-3′, (R) 5′-CAGGTCCTTGCATCACAGCAT-3′, (F) 5′-GTAAAAGAGAGACCCAAGAGCCAG-3′ and (R) 5′-GTAGTCCTTGACAGGTGCTGATG-3′. RT-PCR products were resolved on 10% acrylamide gels and analyzed with a fluorescence imager (LAS-4000; Fujifilm, Minato-ku, Tokyo) after Gel Star staining (Lonza, Bâle, Switzerland). Gene expression levels were measured as the ratio between expression values of the gene of interest and internal 28S rRNA.

### Statistical analysis

Reported data are either reported in a scatter plot where mean values are presented as a horizontal middle line and the SEM by upper and lower horizontal lines or as bar graphs were bars represent mean values and error bars represent SEM.

Statistically significant values were assessed using appropriate tests (Mann-Whitney or *t*-student tests; GraphPad Prism 5). ^*^p<0.05, ^**^p<0.01, ^***^p<0.001.

## SUPPLEMENTARY MATERIALS FIGURES


