# Supplementary material for: Microenvironment-derived ADAM28 prevents cancer dissemination
Source: Oncotarget. 2018 Dec 14;9(98):37185–99. doi: 10.18632/oncotarget.26449 (PMC6324684; doi:10.18632/oncotarget.26449)
Supplement: Supplementary file 1 [file oncotarget-09-37185-s001.pdf]

Microenvironment-derived ADAM28 prevents cancer dissemination

SUPPLEMENTARY MATERIALS

Western blotting

Frozen tissues were crushed into powder using a Mikro Dismembrator S (B. Braun, Biotech International) before protein extraction. Tissues were lysed for 30 minutes in ice-cold lysis buffer (1% Triton X-100, 150 mM NaCl, 1% IGEPAL-CA 630, 1% Na deoxycholate, 0.1% sodium dodecyl sulfate) containing protease inhibitors (Complete, ULTRA Tablets, Mini, EASYpack Protease Inhibitor Cocktail, Roche). Tissue lysates were clarified by centrifugation at 12000 rpm at 4°C for 30 minutes and stored at −20°C. Protein concentrations were determined using the DC Protein Assay kit (Bio-Rad Laboratories, Hercules, CA) and protein samples were resolved on sodium dodecyl sulfate-polyacrylamide gels and subsequently, transferred to polyvinylidene fluoride membranes. Membranes were treated with blocking buffer (PBS with 0.1% Tween-20; Merck, and 1% casein; Sigma Aldrich) for 1 hour at room temperature before overnight incubation at 4°C with rabbit anti-mouse ADAM17 (dilution 1/500, Merck); rabbit anti-mouse ADAM10 (dilution 1/1000, Abcam). Membranes were then incubated for 1 hour at room temperature with swine anti-rabbit/HRP antibody (dilution 1/3000, Dako). Membranes were briefly incubated with Western Lightning Plus-ECL (Perkin Elmer Life Sciences, Boston, MA) according to the manufacturer's instructions. Protein bands were detected using the chemiluminescent imager (LAS 4000; Fujifilm). Subsequent detection of β-actin (rabbit anti-actin; Sigma Aldrich) on the same filters was performed as loading control.

Immunohistochemistry

Deparaffinized 5 µm-tumor bearing lung tissue sections were incubated with rat monoclonal anti-Ki67 (Dako, Glostrup, Denmark) and rabbit anti-cleaved caspase-3 antibodies (Cell signaling, Leiden, Netherlands). Immunostaining was quantified using ImageJ program (NIH, Bethesda, MA, USA).

Flow cytometry

To evaluate apoptosis rates of T lymphocyte populations in WT and ADAM28 KO tissues, following antibodies were used for flow cytometry analysis: CD3-APC CY7 (17A2), CD8-PE (53-6.7), Annexin V V450 and 7-AAD (BD Biosciences). To investigate thymocyte maturation, following antibodies were used: CD8-PE (53-6.7), CD4-PERCP CY 5.5 (RM4-5), CD8a-BB515 (53-6.7), CD44-APC CY7 (IM7) and CD25-BV421 (7D4) (BD Biosciences).

Double negative (DN) thymocyte subsets were gated by CD25 and CD44 expression, defining $CD25^-CD44^+$ as DN1, $CD25^+CD44^+$ as DN2, $CD25^+CD44^-$ as DN3 and $CD25^-CD44^-$ as DN4. Data were acquired on FACS CANTO II flow cytometer (BD Biosciences) and analyzed using BD FACSDiva software (BD Biosciences).

Ex vivo migration assay

Activated $CD8^+$ T cells (2x10^5 cells/200 µl) were suspended in RPMI supplemented with 0.1% BSA and placed in the upper chamber of a 6.5mm transwell insert displaying a 5 µm-pore size membrane (Corning, Corning, New-York). Migration of $CD8^+$ T cells towards increasing CXCL10 concentrations (0, 50, 100, 200 and 400 nM, R&D Systems) present in the lower chamber of the Boyden Chamber was tested. Migration of $CD8^+$ T cells without chemoattractant was determined using serum-free RPMI medium. After 3 hours of incubation, $CD8^+$ T cells, which migrated through the filter, were harvested and stained with anti-CD8a-PE antibody. Activation of $CD8^+$ T cells was confirmed by staining these cells with anti-CXCR3-APC antibody. $CD8^+$ T cells were enumerated in each experimental condition during 1 minute by FACS CANTO II flow cytometer (BD Biosciences). Data were analyzed using BD FACSDiva software (BD Biosciences).

Cytotoxicity assay

$CD8^+$ T cells (100 000 cells) were co-cultured during 2 and 4 hours (37°C) together with 100 000 LLC cells in RPMI supplemented with 10% FBS and 1% Insulin-Transferrin-Selenium (ITS). LLC cells were previously stained with the CellTracker Green CMFDA (Life Technologies) to differentiate these cells from the $CD8^+$ T cells. After the incubation, cells were harvested, washed with PBS/FBS 2% and centrifuged during 6 min at 360 g. Cells were resuspended in 100 µl PBS/FBS 2% and further stained with Annexin V and 7-AAD during 30 minutes at 4°C. Cells were washed twice with PBS/FBS 2% and resuspended in 200 µl PBS prior flow cytometry analysis using FACS CANTO II flow cytometer (BD Biosciences). Data were analyzed using BD FACSDiva software (BD Biosciences).

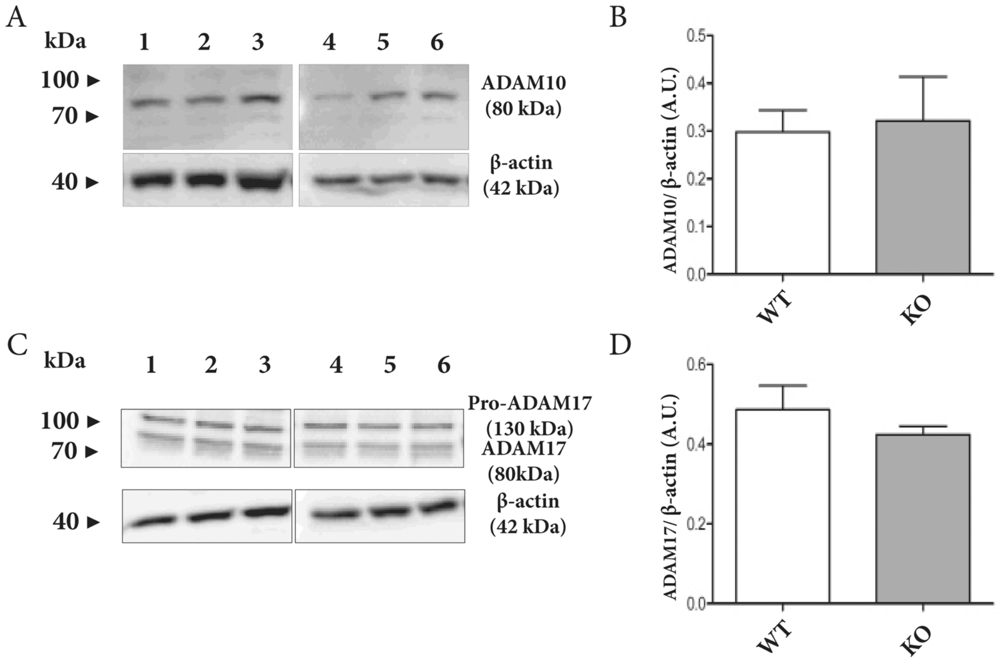

Supplementary Figure 1: No mechanisms are induced to compensate ADAM28 deficiency. (A-C) Western blot analysis measuring ADAM10 (80 kDa) **(A)** and ADAM17 (pro-ADAM17=130 kDa, active ADAM17=80 kDa) **(C)** expression in thymus of tumor-free WT (lanes 1-3) and ADAM28 KO (lanes 4-6) littermates. β-actin production (42 kDa) serves as loading control. **(B-D)** Quantification of ADAM10 and ADAM17 production by scanning densitometry in thymus of tumor-free WT and ADAM28 KO mice (n=3).

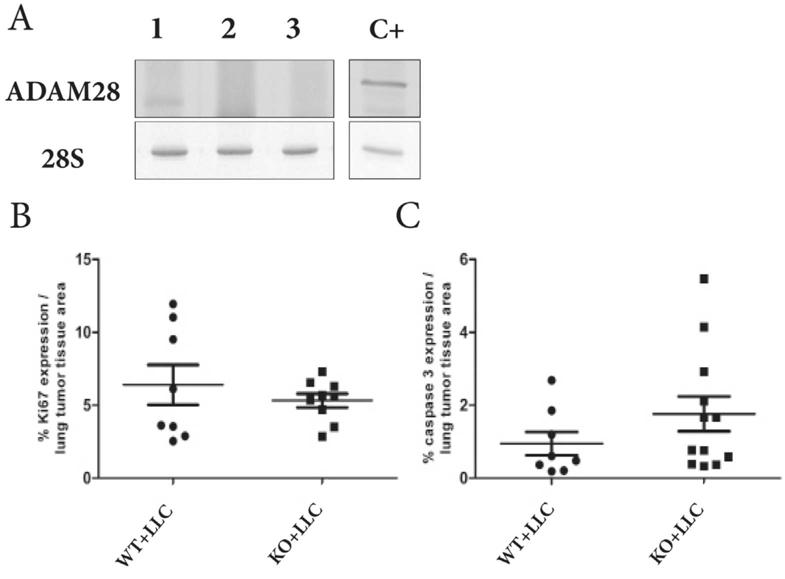

Supplementary Figure 2: ADAM28 deficiency does not impact tumor cell proliferation and apoptosis. (A) mRNA expression levels of ADAM28 in LLC (1), B16K1 (2) and 4T1 (3) tumor cells. mRNA from mouse embryo was used as a positive control (C+). 28S ribosomal RNA levels are shown as loading control. **(B-C)** Quantification of Ki-67 **(B)** and cleaved Caspase-3 **(C)** staining in LLC-derived tumors present in lungs of WT and ADAM28 KO mice.

A

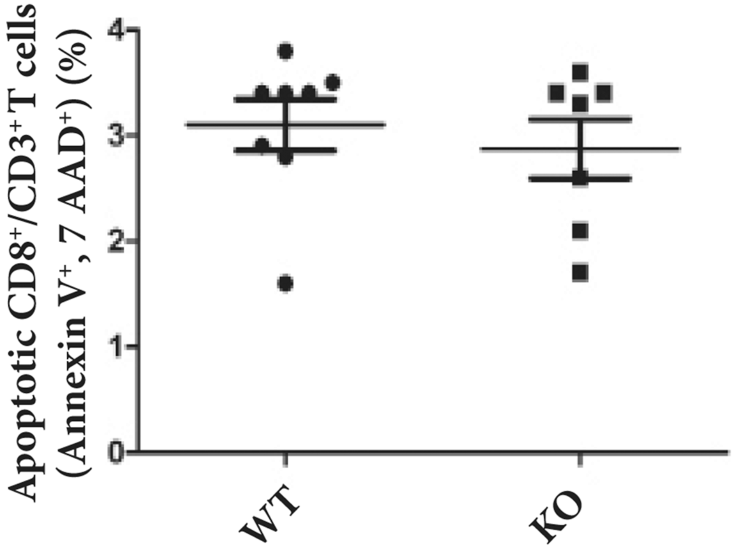

Supplementary Figure 3: ADAM28 deficiency does not influence $CD8^+$ T cell apoptosis. **(A)** Apoptosis rates of $CD8^+$/$CD3^+$ T cells were determined in the spleen of tumor-free WT (n = 8) and ADAM28 KO (n = 7) mice by flow cytometry analysis (Annexin V and 7-AAD staining).

A

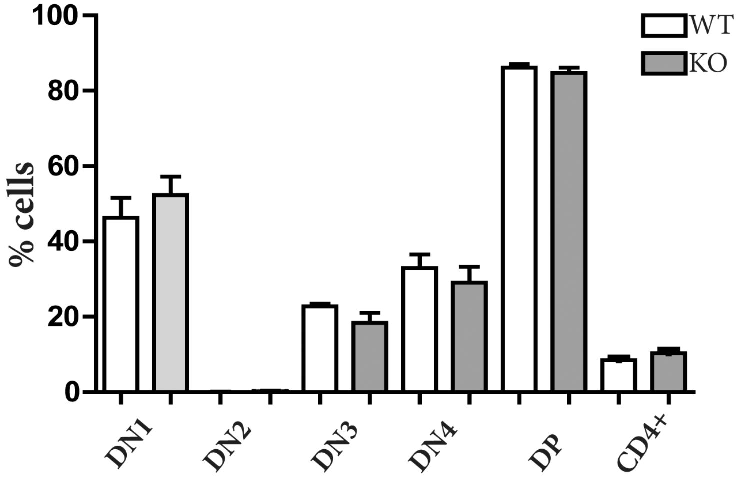

Supplementary Figure 4: ADAM28 deficiency does not influence T cell maturation in thymic tissues. **(A)** Percentages of thymocyte subsets were evaluated by flow cytometry at different stages of T cell maturation (DN1, DN2, DN3, DN4, DP and $CD4^+$ T cells) in the thymus of tumor-free WT (n = 5) and ADAM28 KO (n = 5) mice.

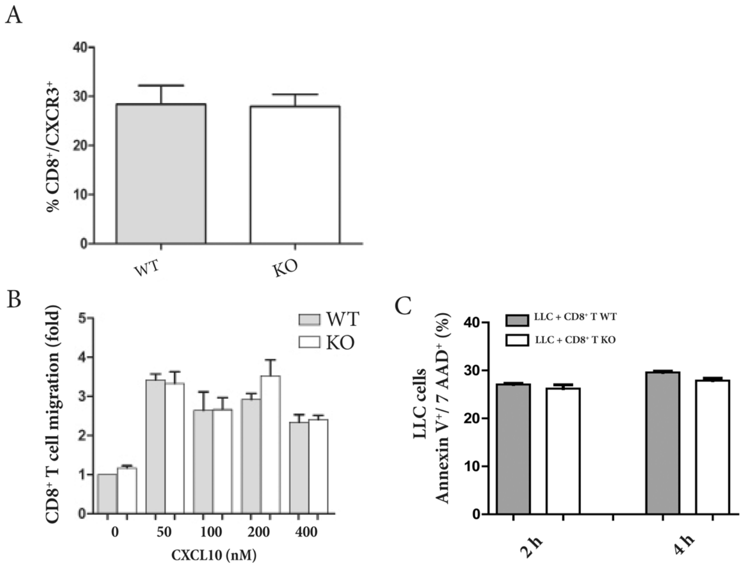

Supplementary Figure 5: Characterization of ADAM28 deficient CD8⁺ T cells. (A) CXCR3 levels measured on the surface of CD8⁺ T cell previously isolated from spleen of WT and ADAM28 KO mice after *ex vivo*-stimulation with IL-2 and IL-7. **(B)** Migration of CD8⁺ T cells was tested in a Boyden Chamber assay where different CXCL10 concentrations (0, 50, 100, 200 and 400 nM) were used as chemoattractants in lower chambers (n=6). All results were normalized to the migration of WT CD8⁺ T cells (without CXCL10 stimulation) considered as baseline migration and expressed as fold increase from baseline. **(C)** *Ex vivo* cytotoxic effects of CD8⁺ T cells isolated from WT and ADAM28 KO mice on LLC tumor cells were measured by flow cytometry 2 and 4 hours after CD8⁺ T cells - LLC co-cultures (Annexin V and 7-AAD staining).
